# Iron Refractory Iron Deficiency Anemia in Dizygotic Twins Due to a Novel *TMPRSS6* Gene Mutation in Addition to Polymorphisms Associated With High Susceptibility to Develop Ferropenic Anemia

**DOI:** 10.1177/2324709617701776

**Published:** 2017-04-19

**Authors:** Joana Pinto, Gustavo Nobre de Jesus, Mónica Palma Anselmo, Lúcia Gonçalves, Daniela Brás, João Madeira Lopes, João Meneses, Rui Victorino, Paula Faustino

**Affiliations:** 1 Universidade de Lisboa, Lisboa, Portugal; 2 Hospital de Santa Maria, Lisboa, Portugal; 3 Instituto Nacional de Saúde Dr. Ricardo Jorge, Lisboa, Portugal

**Keywords:** anemia, refractory, iron, mutation, deficiency

## Abstract

Iron refractory iron deficiency anemia (IRIDA) is an autosomal recessive ferropenic anemia. Its hypochromic microcytic pattern is associated with low transferrin saturation, normal-high ferritin, and inappropriately high hepcidin level. This entity is caused by mutants of the *TMPRSS6* gene that encodes the protein matriptase II, which influences hepcidin expression, an iron metabolism counterregulatory protein. We report two 29-year-old dizygotic female twins with ferropenic, hypochromic microcytic anemia with 20 years of evolution, refractory to oral iron therapy. After exclusion of gastrointestinal etiologies, IRIDA diagnosis was suspected and a novel mutation in the *TMPRSS6* gene was identified. It was found in intron 11 (c.1396+4 A>T) and seems to affect the gene expression. In addition, 3 polymorphisms already associated with a higher risk of developing iron deficiency anemia were also found (D521D, V736A, and Y739Y). Our case reports an undescribed mutation causing IRIDA and supports the hypothesis that this clinical syndrome may be more common than previously thought and its genetics more heterogeneous than initially described.

## Introduction

Iron deficiency anemia is a common entity, encompassing approximately 50% of all anemia cases worldwide.^1^ Its etiology is frequently related with blood loss or gastrointestinal malabsorption.^2^ The increasing number of genetic etiologies related with this anemia such as aceruloplasminemia, ferroportin-1 disease, and hipotransferrinemia requires them to be considered in its differential diagnosis.^3^

Iron refractory iron deficiency anemia (IRIDA) is a hereditary autosomal recessive anemia. Its hypochromic microcytic pattern is associated with low transferrin saturation, normal-high ferritin, and inappropriately high hepcidin level. Contrastingly, ferritin levels are usually within the normal range or high after therapy. Its designation describes an inadequate response to oral iron therapy and an incomplete response to parenteral iron therapy.^4^ The disease is caused by mutations in the *TMPRSS6* gene that encodes matriptase II, a member of the type II transmembrane serine protease family that is expressed in hepatocytes and regulates hepcidin transcription negatively. This entails the fact that hepcidin levels are inappropriately high in correlation to the low levels of free iron in these patients.^5^

We present a case of 2 dizygotic twins with IRIDA and a new mutation located in the intron 11 of the *TMPRSS6* gene (c.1396+4 A>T) that modulates the gene’s splicing in the in silico tests. Additionally, we identified 3 polymorphisms previously associated with a greater risk of developing iron deficiency anemia (SNPs D521D, V736A, and Y739Y).^6,7^

## Case Report

We present two 29-year-old female patients, dizygotic twins. Both have chronic anemia with 20 years of evolution with hemoglobin levels between 8 and 10 g/dL and irresponsive to oral iron therapy. Patient history revealed tiredness with 4 months of evolution, no menstrual disorders or other signs of bleeding abnormalities, and no apparent nutritional deficits. The examination revealed only paleness of the mucosa.

Blood tests revealed anemia with a microcytic and hypochromic pattern and iron deficiency. Laboratory test results are detailed in Table 1. The blood smear revealed anisopoikilocytosis and hipocromia. Anti-DS-DNA, ANA, anti-cytoplasm MPO, and PR3 antibodies were negative. Anti-gliadin (IgG and IgA), anti-transglutaminase (IgA), and anti–*Helicobacter pylori* antibodies were also negative. Hemoglobin A2 and F were normal, and erythroblasts in the peripheral blood were not found. Genetic testing for alpha and beta thalassemia was negative.

**Table 1.** Twins’ Analytic Evaluation at Presentation and Follow-up With Reference Values.

Parameter (Reference Range)	Presentation		Follow-up (6 Months)		Follow-up (18 Months)	
	Twin 1	Twin 2	Twin 1	Twin 2	Twin 1	Twin 2
Hb (12.0-15.3 g/dL)	9.8	8.9	10.2	9.5	10.7	10.5
Hct (36.0-46.0%)	31.6	29.2	32.2	30.0	34.9	33.9
MCV (80.0-97.0 fL)	68.1	64.7	68.0	66.3	75.4	73.2
MCH (27.0-33.0 pg)	21.1	19.7	21.7	20.9	23.2	22.7
MCHC (31.5-35.5 g/dL)	31.0	30.4	31.8	31.5	30.8	31.0
RDW (11.5-14.5 CV%)	18.1	18.5	17.2	17.2	16.4	16.42
Iron (50-170 µg/dL)	19	11	—	—	16	—
TIBC (250-450 µg/dL)	430	395	—	—	—	—
Ferritin (10-291 ng/mL)	6	7	—	—	11	—
Vitamin B_12_ (210-910 pg/mL)	447	524	—	—	515	—
Folates (>5.4 ng/mL)	4.9	5.2	—	—	14.8	24.0
Reticulocytes (%)	2	0.9	—	—	—	—

Abbreviations: Hb, hemoglobin; Hct, hematocrit; MCV, mean corpuscular volume; MCH, mean cell hemoglobin; MCHC, mean corpuscular hemoglobin concentration; RDW, red cell distribution width; TIBC, total iron building capacity.

Upper endoscopy and colonoscopy did not reveal any macroscopic alterations, and biopsies showed conserved architecture and cellular population in the duodenum, distal colon, and rectum. A light intraepithelial lymphocytic infiltrate of the duodenal mucosa is described without atrophy of the villi.

Genetic studies of the *TMPRSS6* gene revealed, in both patients, the presence of heterozygosity of 3 SNPs (single nucleotide polymorphisms), D521D, V736A, and Y739Y, already described associated to higher risk of developing iron deficiency anemia.^6,7^ In addition to these SNPs, a novel mutation corresponding to a substitution (A>T) in the beginning of intron 11 (c.1396+4 A>T) of the *TMPRSS6* gene was also observed. In silico studies (using the Human Splicing Finder software) revealed that this mutation disturbs the intron 11 consensus dador splicing site (wt sequence score = 82.12; mutant sequence score = 73.32; variation −10.72%; MaxEnt −67.34). Consequently, the intron 11 splicing will be affected and the abnormal mRNA will be degraded or translated to a dysfunctional matriptase II protein.

The patients were first submitted to oral iron therapy with 90 mg of ferrous sulfate and 1 mg of folic acid once a day. The evaluation 6 months after revealed remission of the tiredness symptoms. Hemoglobin increased only 0.4 and 0.6 g/dL, respectively. One and a half year after therapy instigation hemoglobin was 1 g/dL higher with concomitant median globular volume and median globular hemoglobin improvement. Serum iron and ferritin also increased but the patients remained anemic.

## Discussion

IRIDA is a hereditary autosomal recessive anemia in which mutations in the *TMPRSS6* gene, which encodes matriptase II, are responsible for hepcidin levels that are inappropriately high in correlation to the low levels of free iron in these patients.^4,5^ Until now, 51 families and 74 patients of different ethical origins have been identified representing a total of 58 different mutations in the *TMPRSS6* gene.^8^ IRIDA’s prevalence is thought to be less than 1:1 000 000,^6^ although it may be underestimated. In some cases, correlation between genotype and phenotype are yet to be defined.^4^

We report an IRIDA case where a novel splicing mutation in the *TMPRSS6* gene was identified together with 3 gene SNPs (D521D, V736A, and Y739Y), already described as being associated with a greater risk of developing iron deficiency anemia.^6,7^ Although IRIDA was first described as an autosomal recessive disease caused by 2 different mutations in the *TMPRSS6* gene (compound heterozygous state) or 2 copies of the same mutation (homozygous state),^9^ there have been increasing reports where this genetic pattern was not encountered but where patients presented with the clinical manifestations and course of IRIDA.^8^ In agreement, Nai et al showed that mice with just one *TMPRSS6* mutation had greater predisposition for the development of anemia comparatively with mice without any mutations.^10^

In this case, according to the information obtained during the in silico testing, the location of the novel *TMPRSS6* gene mutation affects the gene’s splicing. Consequently, the abnormal mRNA will be degraded or translated into a shorter and nonfunctional matriptase II protein. In addition to this pathogenic mutation, the 2 patients present heterozygosity for 3 SNPs already associated with high risk of developing iron deficiency anemia. The genetic combination of this likely pathogenic mutation with the 3 functional SNPs seems to reduce matriptase II effect to an insufficient level that promotes IRIDA development. Our patients’ unusual *TMPRSS6* genotype, the clinical history, and the weak response to oral iron therapy resemble other IRIDA cases.^6,8,10,11^ For example, Pellegrino et al demonstrated in a family case study that one *TMPRSS6* gene mutation in combination with 2 or more SNPs can clinically present as IRIDA.^11^

Iron refractory anemia is defined as a weak, slow, and incomplete response to oral iron therapy, with an inadequate improvement of hematological parameters.^12^ Although most authors do not attempt a strict definition, Camaschella et al defined iron refractory anemia as an improvement lower than 1 g/dL of hemoglobin after 4 to 6 weeks of therapy with an equivalent of 100 mg/day of elemental iron.^13,14^ Having excluded other causes of iron deficiency, this analytical finding associated with the iron deficiency pattern characteristic of IRIDA (low transferrin saturation, normal-high or normal ferritin, and inappropriately high levels of hepcidin) and family history of IDA should prompt the possibility of this diagnosis.^5^ In the cases reported here, after 18 months of oral iron therapy the twins’ hematological parameters did not improve as expected and they remained anemic (hemoglobin 10.7 and 10.5 g/dL) and ferropenic, with low serum iron (16 µg/dL) and ferritin in the lower normal limit (11 ng/mL), confirming the inadequate response to treatment typical of IRIDA patients.

Hepcidin reference values for healthy individuals have not yet been established. Bregman et al reported that hepcidin levels could predict oral iron response in patients with IDA. Hepcidin levels greater than 20 ng/dL represented an 81.6% probability of a nonresponse to oral iron therapy and two thirds of these patients responded to parenteral iron therapy.^15^ The fact that IRIDA’s diagnosis can only be confirmed through genetic testing and that hepcidin assay has yet to be standardized led to the decision of not doing such analysis. We acknowledge the possibility that the use of a standardized hepcidin assessment is an indicator of a possible resistance to oral iron therapy.

Currently, parenteral iron is the most viable therapeutic option for IRIDA patients. New intravenous iron formulations (ferric gluconate und iron sucrose) present less adverse effects and a quicker response comparatively to high-molecular-weight iron dextran to which hypersensitivity reactions are frequent.^13^ Additionally, parenteral iron can be directly transported to erythropoietic precursors trough transporters like transferrin, bypassing the reticuloendothelial system.^16^ We conclude that this therapeutic option should be considered regarding its benefits and possible risks in patients with slow and weak response to oral iron therapy. Cau et al presented a case of a 5-year-old female child with a single *TMPRSS6* gene mutation refractory to oral iron therapy that responded to oral iron supplemented with ascorbic acid.^17^ Although greater evidence of the resourcefulness of this therapy is needed, this alternative might be considered in patients where parenteral iron therapy is contraindicated. Even though recombinant eritropoietin therapy has shown promising results in patients with chronic anemia, it did not increase hemoglobin levels or hepcidin clearance in patients with IRIDA.^18^

In our case, IRIDA was suspected after an inadequate response to oral iron therapy. This highlights the necessity of establishing universally accepted criteria for an inadequate response to oral iron therapy. This case also supports the hypothesis that the clinical syndrome that accompanies IRIDA might be more common than previously thought and its genetics more heterogeneous than initially described. We agree with other authors’ views that IRIDA should be more widely considered in the differential diagnosis of iron deficiency anemia.^19^ Moreover, hepcidin routine measurement can also be helpful for the differential diagnosis between iron deficiency anemia and IRIDA. Nevertheless, *TMPRSS6* genetic study remains crucial, enabling not only IRIDA’s definitive diagnosis but also further characterizing the disease’s genetic and clinical correlation.
